# Ambulatory Surgery Center in Rural Uganda: A Novel Approach to Providing Surgical Care

**DOI:** 10.7759/cureus.55848

**Published:** 2024-03-09

**Authors:** Unwana Abasi, Joseph Okello Damoi, Anna Turumanya Kalumuna, Angellica Giibwa, So Park, Dylan Cuva, Allen T Yu, Arthur Emoru, Moses Bakaleke Binoga, Dillan Villavisanis, Sara N Kiani, Katie Glerum, Jerome Waye, Michael Marin, Linda Zhang

**Affiliations:** 1 Surgery, Icahn School of Medicine at Mount Sinai, New York, USA; 2 Surgery, Kyabirwa Surgical Center, Jinja, UGA; 3 Surgery, Mount Sinai Hospital, New York, USA; 4 Anesthesiology, Kyabirwa Surgical Center, Jinja, UGA; 5 Plastic and Reconstructive Surgery, Perelman School of Medicine at the University of Pennsylvania, Philadelphia, USA; 6 Gastroenterology, Mount Sinai Hospital, New York, USA

**Keywords:** rural surgery, uganda, effectiveness, disability-adjusted life years, ambulatory surgery

## Abstract

Background: Despite evidence that ambulatory surgery is safe with faster recovery compared to in-patient hospitalization, surgeons in low- and middle-income countries like Uganda have been hesitant to embrace this practice. Kyabirwa Surgical Center (KSC) is the first freestanding ambulatory surgery center (ASC) in rural Uganda. We aim to report the impact of a rural ASC since its establishment, in alleviating surgically-treatable morbidity within its catchment area.

Methods: KSC is located in Jinja, Uganda. The center’s electronic medical record was used to analyze the utilization of services, and the Uganda Bureau of Statistics was used to calculate KSC’s catchment area. Effectiveness was calculated using disability-adjusted life years (DALYs) averted.

Results: Between July 2019 and December 2021, 7,391 patients (57.7% female, 42.3% male) visited KSC from a catchment area of 570,790 people. Of 1,355 procedures, 64.6% were general surgery, 21.3% endoscopy, 9.2% gynecological/genitourinary), 2.8% ENT, 1.5% colorectal, and 0.6% orthopedics. There were no postoperative hospital admissions for complications or mortalities. From the seven most common procedures with an associated disability weight, 2,193.16 total DALYs were averted.

Conclusion: ASCs can be effective in addressing surgical care gaps in Uganda by increasing the yearly surgical capacity of the local catchment area and averting DALYs within the population.

## Introduction

The Lancet Commission estimates that five billion people worldwide cannot access safe and affordable surgical and anesthesia care [1]. Historically, public health efforts for low- and middle-income countries (LMICs) have targeted communicable diseases and maternal and neonatal mortality, as targeting surgery was often considered too resource-intensive [2,3]. However, with 11% of global disability-adjusted life years (DALYs), plus the associated macroeconomic impact, attributable to surgical and anesthesia needs, the necessity for surgical services is clear [4,5]. In Uganda specifically, 241 procedures are performed yearly per 100,000 population, far less than the 5,000 procedures recommended by the Lancet Commission.

Most surgical care in LMICs is addressed through inpatient admission. In Uganda, patients are usually admitted the day before low-acuity surgery and discharged several days postoperatively. These inpatient days increase the risk for complications, including hospital-associated infections, adverse drug reactions, antibiotics over-prescription, and immobility-related complications [6,7]. Prolonged hospitalization also increases the cost of care for both patients and facilities, which is particularly problematic in LMICs where resources are limited and healthcare expenditure can result in significant economic impoverishment for the patient [8-10].

By contrast, the standard of practice in high-income countries (HICs) has transitioned towards ambulatory procedures, yielding major advantages for patients and the health system, such as lower odds of readmission for low-acuity procedures [11]. Furthermore, ambulatory surgery is comparable to inpatient stay with regard to patient safety and acceptability [12-14]. Despite strong evidence in HICs suggesting that ambulatory surgery is safer and more cost-effective compared to hospitalization, it has not achieved widespread practice in LMICs due to concerns regarding postoperative healthcare infrastructure and the safety of day surgery [15].

Kyabirwa Surgical Center (KSC) in Jinja, Uganda, is the first freestanding facility to provide exclusively ambulatory surgeries to the surrounding rural population. The facility aims to replicate the advantages of ambulatory surgery proven in HICs and demonstrate that ambulatory surgery centers (ASCs) can be an effective addition to the healthcare system in order to address the gap in surgical care in LMICs. There is limited literature on the adoption of ambulatory surgery in LMICs, and none examining freestanding surgery centers that focus on ambulatory cases in rural communities [16,17]. Here we present the demographics, most common procedures, and calculated effectiveness of establishing an ASC in rural Uganda.

## Materials and methods

Setting

Kyabirwa Surgical Center, located in Kyabirwa village in the Jinja district of Eastern Uganda, was designed as a replicable, self-sustaining surgical facility, utilizing solar paneling for electricity and a rainwater harvesting system to tap rainwater. Collaborating with regional architects and local contractors ensured compliance with local customs, laws, and health regulations while supporting the local economy. There are two operating rooms, six recovery beds, a clinical laboratory with pathology services, and a radiology room for diagnostic ultrasounds and X-rays. Consultation visits at KSC began in July 2019, and the first surgery was performed in September 2019. To supplement postoperative care, KSC has utilized a home-based program for follow-up of non-endoscopic procedures. A nurse calls patients on postoperative days 1, 7, and 21 to inquire about postoperative symptoms and performs an at-home visit on postoperative day 3 to assess the patient’s symptoms and the wound.

The entirely Ugandan staff is comprised of one surgeon, two anesthesia providers, two medical officers, and nine nurses providing clinical care. Remote consultation is also available if requested by KSC, which facilitates inter-institutional learning, enhancing the knowledge of all parties [18]. A substantially discounted fee is charged for surgical procedures, with further discounts or free surgery available upon discussion with KSC’s psychosocial department.

Medical records were captured using ClinicMaster, an electronic medical record developed by a Ugandan software development company, ClinicMaster INTERNATIONAL (Kampala, Uganda). Deidentified patient data from ClinicMaster were summarized in Microsoft Power BI (Microsoft Corporation, Redmond, Washington, United States), and the data collected between July 2019 and December 2021 were analyzed for this study.

Calculating effectiveness

The 10 most common procedures performed were considered to measure effectiveness, which was calculated in terms of DALYs averted. Only surgeries associated with a diagnosis and disability weight (DW) recognized by the Global Burden of Disease (GBD) were included in the measurement. Adenotonsillectomy, esophageal stenting, and orchiopexy were excluded due to lack of a DW, and so the seven procedures included in the total DALYs calculation included hernia repair, abscess incision and drainage, biopsy, circumcision, appendectomy, hydrocelectomy, and thyroidectomy. A simplified version of the DALYs calculation, first applied towards emergency obstetric care in Bangladesh and now commonly used in cost-effectiveness analysis literature, was utilized in this study [19,20].

Each intervention was assigned a score for risk of death/disability (severity of the condition) and effectiveness of treatment estimates based on probability figures used in previous literature [21]. Years of life lost/lived in disability were calculated using the “ideal” life expectancy of 82.5 years for females and 80 years for males. For simplification, an average of 81.25 years was used for all patients. DWs from the 1990 and 2010 GBD studies were used when available [22]. For the procedures that did not have an associated DW, the GBD initiative recommendation of referring to “generalized illnesses” was used, as well as expert opinions. For example, appendicitis was given a DW of 0.463, which is comparable to the listed “severe abdominopelvic problem” in the GBD database. In brief, DALYs averted = YLD/YLL x RD x DW x PST, where YLD/YLL is Years of life lived in disability/years of life lost, RD is Risk of death, and PST is the probability of successful treatment.

DALY calculations were made without age weighting or discounting. To assess the potential future impact of KSC, a regional catchment area was calculated, described below. Uganda-specific prevalence rates of each procedure were taken from the 2019 Global Health Data Exchange to estimate the possible DALYs averted if KSC’s entire catchment area were treated.

Calculating catchment area

The catchment area was calculated using patient visit data from July 2019 to September 2021 and 2014 census data from the Uganda Bureau of Statistics (UBOS). Patient addresses were extracted from ClinicMaster, and a parish or town was assigned to each address using the Land Conflict Mapping Tool, which divides regions of Uganda to the village level. The parishes and towns contributing to ≥ 0.5% of KSC’s patients were extracted, and a population was assigned to each according to UBOS 2014 census data and citypopulation.de, a population statistics site that obtains data from UBOS [23]. District-specific projection data from UBOS were then used to estimate 2021 population sizes, which were summed to determine catchment size.

## Results

Patient demographics

As of December 2021, 7391 patients (57.7% female, 42.3% male) visited the facility. Patient records up to September 2021 showed that 56.2% of patients were from the nearby Jinja District. The catchment area of the center, based on patient visits through September 2021, was estimated to be 570,790 patients.

Labs/imaging

A total of 3,174 lab tests were performed at KSC, including 1,863 preoperative laboratory tests, 593 infectious disease screenings, and 260 cancer screenings. There were 3,281 ultrasound studies, primarily abdominopelvic scans (69.1%), obstetrics and gynecology studies (12.6%), and soft tissue ultrasounds (12.5%). A total of 920 radiographs were completed, most commonly X-rays of the chest (45.2%), extremities (26.8%), and spine (19.2%) (Table 1).

**Table 1:** Breakdown of Investigations at Kyabirwa Surgical Center Data is presented as n (%). ^a ^Includes urinalysis, d-dimer, stool analysis, urine hCG, and bleeding time; ^b^Includes ultrasounds of the head, neck, thyroid, breast, and chest^; c^Includes barium swallow in imaging of the thoracic inlet and scapula. STD: sexually transmitted disease; OB-GYN: obstetrics and gynecology; hCG: human chorionic gonadotrophin

Diagnostic tests/imaging	Frequency (Percentage), n (%)
Laboratory Tests (N=3174)	
Pre-Operative & Basic Labs	1863 (58.7)
Infectious Disease Screening	592 (18.7)
Cancer Screening	260 (8.2)
Endocrine Workup	183 (5.8)
STD Screening	98 (3.1)
Other^a^	179 (5.6)
Ultrasound (N=3281)	
Abdominal/Pelvic	2268 (69.1)
OB/GYN	415 (12.6)
Soft Tissue^b^	410 (12.5)
Testicular/Scrotal	157 (4.8)
Orthopedic	19 (0.6)
Vascular	12 (0.4)
X-ray (N=920)	
Chest	416 (45.2)
Extremity	247 (26.8)
Spine	177 (19.2)
Abdominal	32 (3.5)
Pelvis	23 (2.5)
Skull/Facial Bone	14 (1.5)
Other^c^	11 (1.2)

Types of procedures performed

During the study period, 1,355 procedures were performed (Table 2). Of these procedures, 64.6% were within general surgery, 21.3% were endoscopies, 9.2% in gynecological/genitourinary, 2.8% in ENT, 1.5% in colorectal surgery, and 0.6% in orthopedics. The most common procedures included 356 (26.3%) hernia repairs, 214 (15.8%) upper GI endoscopies, 189 (13.9%) soft tissue tumor excisions, and 117 (8.6%) biopsies. The modes of anesthesia included general (36.6%), spinal (23.4%), local (22.7%), and monitored sedation (15.6%). The patients were monitored in the recovery room and then discharged once awake, alert, and ambulating, with pain well managed on oral analgesics.

**Table 2:** Breakdown of Surgical Procedures Performed at Kyabirwa Surgical Center Data presented as n (%) ^a^Includes exploration of a superficial foreign body, ingrown nail excision, foreign body removal, fundoplication, contracture release, diagnostic laparoscopy, and digital fission; ^b^Includes esophageal stenting and percutaneous endoscopic gastrostomy; ^c^Includes genital wart removal, labial separation, myomectomy, hymenectomy, suprapubic catheter placement, and scrotal exploration; ^d^Includes foreign body removal, thyroglossal duct cyst excision, nasal endoscopy, tongue tie release, ear syringing, and sinus exploration. FNA: fine needle aspiration; GYN/GU: gynecological/genitourinary

Procedures	Frequency (Percentage)
General Surgery (N=896)	
Hernia	356 (39.7)
Soft Tissue	189 (21.1)
Wound Care	137 (15.3)
Biopsy/FNA	123 (13.7)
Appendectomy	26 (2.90)
Colorectal	20 (2.32)
Amputation	9 (1.00)
Mastectomy/Lumpectomy	5 (0.558)
Exploratory Laparotomy	4 (0.446)
Biliary	2 (0.223)
Other^a^	25 (2.79)
Gastroenterology (N=288)	
Esophagogastroduodenoscopy	214 (74.3)
Colonoscopy	63 (21.9)
Other^b^	11 (3.82)
GYN/GU (125)	
Circumcision	68 (54.4)
Hydrocelectomy	23 (18.4)
Orchiopexy/Orchiectomy	15 (12.0)
Varicocelectomy	4 (3.20)
Vasectomy	2 (1.60)
Other^c^	13 (10.4)
ENT (N=38)	
Thyroidectomy	13 (34.2)
Tonsillectomy	10 (26.3)
Laryngoscopy	2 (5.26)
Other^d^	13 (34.2)
Orthopedic (N=8)	
Total	1355

Postoperative visits

Between June 2020, when the home visitation program began, and November 2021, a nurse performed 572 home visits, finding no hospital admission for postoperative complications and no postoperative mortalities. The nurse called the surgeon for further assistance on home visits 10% of the time. Based on the surgeon’s remote assessment, no patients were advised to return urgently to the clinic.

Effectiveness of the ASC

The seven procedures included in the total DALYs calculation comprised 49.8% of the procedures. A total of 2,193 DALYs were averted between September 2019 and December 2021 (Table 3). Hernia repair and appendectomies represented 28.2% of the cases, yet made up 80.5% of DALYs averted. Table 4 shows the projected DALYs that could be averted if the entire catchment area of 570,790 people were treated. The total projected DALYs averted was 585,639.59, with 462,434.06 (79.0%) DALYs attributable to circumcision and 109,519.33 (18.7%) attributable to incision and drainage.

**Table 3:** Scores and DALYs Averted for Surgical Procedures at Kyabirwa Surgical Center, September 2019-December 2021 ^a^Includes mesh repair, herniorrhaphy, herniotomy, and Mayo repair; ^b^Includes punch biopsy, tru-cut biopsy, incisional biopsy, and excisional biopsy. DALYs: disability-adjusted life years

Procedure	No. of patients	Disability weights	Severity Score	Effectiveness Score	Average DALYs averted per case	Total DALYs averted
Hernia repair^a^	356	0.3	0.3	0.8	2.996	1066.576
Abscess Incision & Drainage	72	0.108	0.1	1	0.656	47.232
Biopsy^b^	117	0.294	0.3	0.8	2.435	284.895
Circumcision	68	0.151	0.1	1	1.157	78.676
Appendectomy	26	0.463	1	1	26.86	698.36
Hydrocelectomy	23	0.123	0.1	0.8	0.348	8.004
Thyroidectomy	13	0.199	0.1	1	0.725	9.425
Total	675	-	-	-	-	2193.168

**Table 4:** Projected DALYs That Could be Averted for Surgical Procedures at Kyabirwa Surgical Center ^a^Prevalence rate taken from GBD database specific to Uganda for individual procedures. ^b^Using a catchment area of 570,790 people including areas with at least 0.5% of study hospital patients from July 2019-September 2021. DALYs: disability-adjusted life years; GBD: Global Burden of Disease; I&D: incision and drainage

Procedure	Prevalence rate^a^	Average DALY averted per case	Projected number of patients^b^	Projected DALYs averted
Hernia repair	0.30%	2.996	1712	5130.61
Abscess I&D	29.00%	0.656	167013	109519.326
Biopsy	0.26%	2.435	1484	3613.293
Circumcision	70.00%	1.157	399553	462434.055
Appendectomy	0.01%	26.86	57	1533.13
Hydrocelectomy	0.30%	0.348	1712	595.457
Thyroidectomy	0.68%	0.725	3881	2813.717
Total	-	-	575412	585639.591

## Discussion

KSC opened in 2019 to provide safe, affordable surgery to patients in rural Eastern Uganda. Between July 2019 and December 2021, KSC received 7,000 patients, 56.2% from the local Jinja district. These visits resulted in over 3,100 laboratory and 3,200 imaging investigations, and over 1,300 procedures in 28 months. With a baseline of 241 procedures performed yearly per 100,000 population in Uganda, this value amounts to 1,355 surgical procedures per year within the center’s 570,790-person catchment area.

Through seven of the most common therapeutic interventions, 2,193 DALYs were averted. This DALY amount is small compared to values seen in the literature [20,24], and is comparable to the global health burden of Leishmaniasis infection (2,357 DALYs). However, compared to the large inpatient hospitals typically examined, KSC is a small surgical center focusing on elective procedures associated with smaller DALYs values. Expansion into procedures associated with higher DALYs values will provide a more accurate assessment of KSC’s ability to impact DALYs caused by surgical conditions within the local region. Additionally, the 2,193 DALYs averted account for only seven of the most common procedures, and the model used simple DWs that do not account for the severity of the condition. Further exploration into the types and the severity of the procedures performed will give a more accurate understanding of the morbidity and mortality averted through KSC’s surgeries, likely with an increased DALYs averted value. Additionally, incorporating factors such as age-weighting and discounting will improve the assessment of KSC’s effectiveness.

This study only begins to demonstrate the value of an ambulatory surgical center to the local community. Visiting patterns for KSC have shown an average of 103 new patients per month in 2019, followed by 221 per month in 2020 and 353 per month in 2021. These values, achieved by a growth in the clinical staff over the years, represent an encouraging trend towards this ASC meeting the surgical needs of the population in its catchment area. An ASC also widens access to clinical diagnostic studies, evidenced by the clinical studies performed for non-surgical patients who need affordable investigations to guide their outside physician. For instance, 391/3281 of ultrasounds (12%) were for obstetric patients. Additionally, 910/3174 (28.7%) laboratory tests and 141/920 (15.3%) x-rays were performed for nonsurgical patients at the request of their physicians.

Adoption of the ASC model has been slow in LMICs because of healthcare providers’ fear for patient safety. There is concern that a rural setting may make it difficult to access emergency postoperative care, and patients or their families may not perform postoperative care [16,17,25]. KSC carefully screens which procedures and patients are appropriate for an ASC and supports an ambulance service in case of unforeseeable events that require expedient transfer. Moreover, there is a stringent postoperative follow-up algorithm, and patients are counseled on postoperative care and expectations. Thus far, no intraoperative complications have led to patient transfer, and there have been no mortalities related to treatment at KSC.

Despite the benefits of a facility such as KSC, limitations exist in this model. The cost of building, running, and maintaining an ASC that provides surgical services in a rural setting requires significant capital and consistent funding to remain financially viable. Philanthropic donations were suitable for start-up costs, but financial self-sustainability is paramount for the facility to continue providing low-cost surgeries and for the replicability of the model. Another concern is that low surgical workforce capacity limits scalability and makes it difficult to justify shifting human resources toward elective ambulatory procedures [26,27]. Task-shifting surgical care to trained non-surgeon clinicians such as medical officers and field nurses is a known alternative to bolster workforce capacity and is currently employed at KSC [28-30].

## Conclusions

More exploration is needed to further support the safety and scalability of the ASC model. Future studies will examine the effect of KSC in improving patient outcomes, decreasing the length of stay, and improving cost-effectiveness for patients and the facility. Analyzing KSC’s impact on the community can potentially reveal a reduction in barriers to accessing surgery, acceptability within the community, and a decrease in the financial impact of surgery on the patient population. Taken together, the preliminary results of this study show that rural ASCs may be an effective approach to addressing the gap in surgical care in Uganda by serving the basic surgical needs and averting DALYs within a rural population.
